# Trait impulsivity influences behavioural and physiological responses to threat in a virtual environment

**DOI:** 10.1038/s41598-024-60300-6

**Published:** 2024-04-25

**Authors:** Christopher Baker, Stephen Fairclough, Ruth S. Ogden, Rachel Barnes, Jessica Tootill

**Affiliations:** 1 School of Computer Science & Mathematics, Liverpool John Moores University, Liverpool, UK; 2 School of Psychology, Liverpool John Moores University, Liverpool, UK

**Keywords:** Psychology, Computer science

## Abstract

Trait impulsivity represents a tendency to take action without forethought or consideration of consequences. This trait is multifaceted and can be decomposed into attentional, motor and non-planning subtypes of impulsivity. The purpose of the current study was to investigate how subtypes of trait impulsivity responded to different degrees of threat within room-scale virtual reality (VR) with respect to behaviour and level of physiological activation. Thirty-four participants were required to negotiate a virtual environment (VE) where they walked at height with the continuous threat of a virtual ‘fall.’ Behavioural measures related to the speed of movement, interaction frequency and risk were collected. Participants also wore ambulatory sensors to collect data from electrocardiogram (ECG) and electrodermal activity (EDA). Our results indicated that participants who scored highly on non-planning impulsivity exhibited riskier behaviour and higher skin conductance level (SCL). Participants with higher motor impulsivity interacted with more objects in the VE when threat was high, they also exhibited contradictory indicators of physiological activation. Attentional impulsivity was associated with a greater number of falls across the VE. The results demonstrate that subtypes of trait impulsivity respond to threats via different patterns of behaviour and levels of physiological activation, reinforcing the multifaceted nature of the trait.

## Introduction

Impulsivity is a multifaceted trait characterised by a predisposition to act hastily and without adequate forethought, which can result in negative consequences for the individual or others^1,2^. Impulsive behaviour is often perceived to be characteristic of youth and is expected to diminish as people mature. In mature individuals, impulsivity is generally considered to be a maladaptive trait. Empirical evidence has linked trait impulsivity to substance abuse^3–6^, aggressive tendencies^7^, pathological gambling^5^ and criminal behaviour^8,9^. High levels of trait impulsivity has also been associated with career difficulties^10^, marital strife^11^, overeating^12,13^ and risky sexual choices^14,15^, as well as psychiatric disorders e.g. ADHD (Attention Deficit Hyperactivity Disorder)^16,17^, BPD (Borderline Personality Disorder)^18,19^, ASPD (Antisocial Personality Disorder)^20^, bipolar and schizophrenia conditions^21,22^.

In the psychological literature, impulsivity refers to personality traits and behaviours that are inherently multifaceted^23^. The trait of impulsivity is characterised as a tendency to take action without consideration of consequences^24,25^; it also exhibits some overlap with the trait of sensation seeking^26^, e.g., disinhibition, susceptibility to boredom. Behavioural manifestations of impulsivity include an inability to inhibit an inappropriate action or response, which is called motor impulsivity^24,27,28^. Impulsive individuals tend to commit errors as a direct consequence of their tendency to make fast decisions without gathering and evaluating information^29^ (reflection impulsivity); similarly, they may have difficulties in delaying reward gratification^30–32^, known as temporal impulsivity^33^. These cognitive characteristics may stem from a fundamental attentional deficit as impulsive behaviours may reflect an inability to focus or concentrate on relevant information^24^; see Herman et al.^23^ and Winstanley et al.^31^ for summaries.

A number of variables are known to modulate both trait and behavioural forms of impulsivity. These factors can include demographic characteristics, such as gender^34,35^ and age^36^. However, psychophysiological arousal and affective processes relating to emotional reactivity also can also modulate impulsivity, enhancing the tendency towards rash action^25^, e.g., non-planning or motor impulsivity^24^. Psychophysiological activation has been associated with both decision-making^37^ and behavioural impulsivity in a number of ways. The under-arousal hypothesis (Eysenck and Eysenck^38^) argued that individuals with lower baseline arousal exhibit greater propensity to seek risk and other forms of stimulation^38^. Similarly, Damasio and colleagues outlined a neurobiological framework^39–42^ where decision-making processes in the prefrontal cortex are influenced by ‘somatic markers' and activation at an autonomic level. With respect to empirical evidence, lower baseline arousal has been associated with increased behavioural impulsivity^43^, whereas Mathias & Stanford reported under-arousal in impulsive individuals at rest, which transformed into higher activation for those individuals under conditions of challenge^44^. When levels of physiological arousal were pharmacologically increased, the tendency towards temporal impulsivity was reduced while reflection impulsivity was enhanced^45^. There is also evidence that reduced interoceptive sensitivity (cardiac discrimination) is associated with increased trait scores on non-planning impulsivity^45^, which is broadly supportive of the somatic marker hypothesis, i.e., impulsivity is associated with blunted awareness of autonomic activation with consequences for decision-making and behaviour. However, others have found no evidence of any association between impulsivity and autonomic activity from electrodermal activity (EDA) or heart rate during a gambling scenario^45^.

Levels of physiological arousal and trait impulsivity can both interact to influence how an individual perceives and responds to threat-related stimuli. The presence of threat tends to increase behavioural impulsivity, reducing response inhibition^46^ and decreasing reaction time^47^. This tendency to act quickly and frequently without fully evaluating a situation is reinforced by intense emotional experiences, such as anxiety^48,49^. There is also evidence that an increased level of physiological arousal associated with intense emotions can serve as the primary driver of impulsive behaviour^50,51^. However, given the multifaceted nature of trait impulsivity and the multidimensional measurement of physiological activation, the nature of how both variables interact to influence responses to threat remains complex and unclear.

The purpose of the current study was to delineate the relationship between subtypes of trait impulsivity, psychophysiological arousal, and behavioural responses to threat in virtual reality (VR). A large-scale virtual environment (VE) was utilised to induce intense emotional experiences^52^ by creating a sense of presence^53^ and proximity to virtual threat in a way that enhanced ecological validity. Our VE also permitted a high degree of agency in participants to elicit individual differences in behaviour while manipulating threat in a controlled environment^54–58^. Our virtual environment (VE) induced threat by creating an illusion of height and manipulating the probability of a virtual ‘fall’^59^. Participants were empowered to take agentic actions through the incorporation of natural sensorimotor contingencies as they navigated the VE. A pivotal element of the task's design was to foster strategic behaviour, thereby enhancing both agency and ownership over actions. This approach was designed to prevent frustration and disengagement, even as the level of threat or difficulty was systematically escalated as participants progressed through the course.

The current study required to negotiate a route through a large-scale (13.6 × 8.4 m) VE while equipped with wearable sensors to measure EDA and heart rate. Our primary research questions were: (1) do subtypes of trait impulsivity lead to similar or different patterns of behaviour in response to a high level of threat, and (2) to what extent are changes in autonomic arousal in response to threat predicted by subtypes of trait impulsivity and behavioural measures. Trait impulsivity was assessed using the Barratt Impulsiveness Scale (BIS-11)^24^. We anticipated that impulsive individuals (particularly motor and non-planning subtypes) would respond to high levels of threat by failing to adjust behaviour, i.e., because they tend to act without thought. We also predicted that physiological activation would modulate behaviour when threat is high and sustained.

## Methods

### Participants

Thirty-four participants (14 female) were recruited to the study from a university population of undergraduates and postgraduates. The mean age of the participants was 23 years (SD = 3.5 years). The only criterion for inclusion was that participants must be aged over 18 years and able to walk without assistance. The experimental protocol was approved by the Liverpool John Moores University Research Ethics Committee prior to data collection (Ref: 22/PSY/007), which operates within guidelines from the UK Research Integrity Office Code of Practice for Research and in accordance with the Declaration of Helsinki.

### Virtual environment

**Figure 1:**
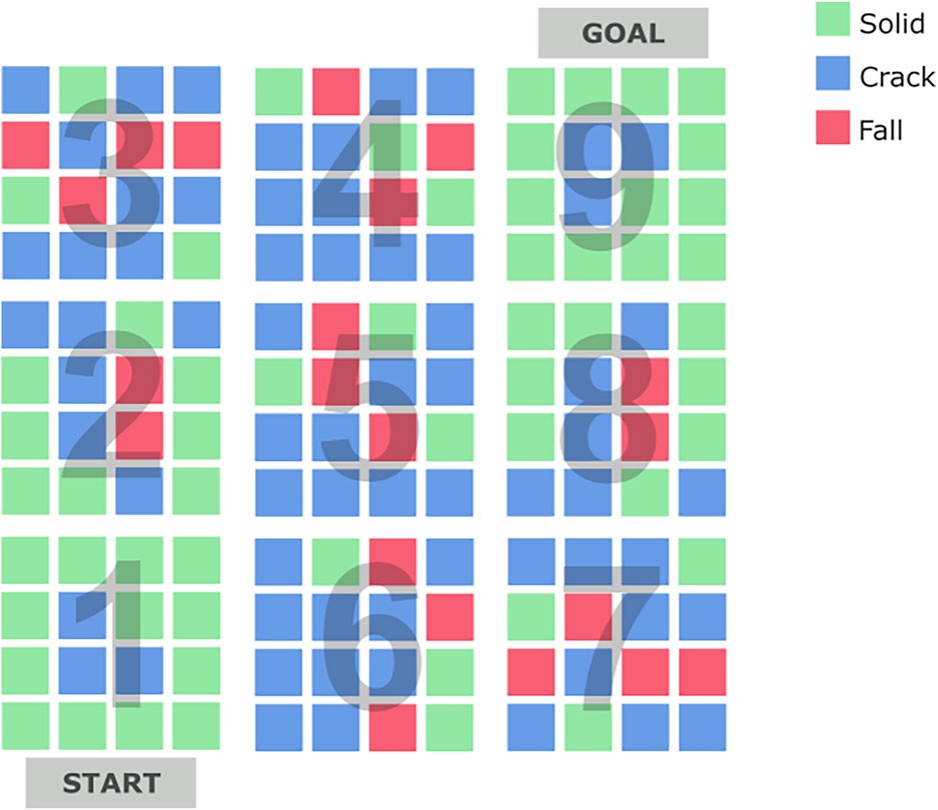
Layout of the virtual environment.

Participants were required to negotiate a path across a large grid of ice blocks suspended in the air at a virtual height of 200 m (see Fig. 5). The layout of the VE is illustrated in Fig. 1. Participants were required to follow the route from section 1 to section 9 in the numbered sequence shown in Fig. 1. The end goal of the VE was represented by a door that must be activated by hand to leave the VE. The VE occupied a physical space of 13.6 × 8.4 m, and each individual block shown in Fig. 1 was approximately 70 × 70 cm.

**Figure 2:**
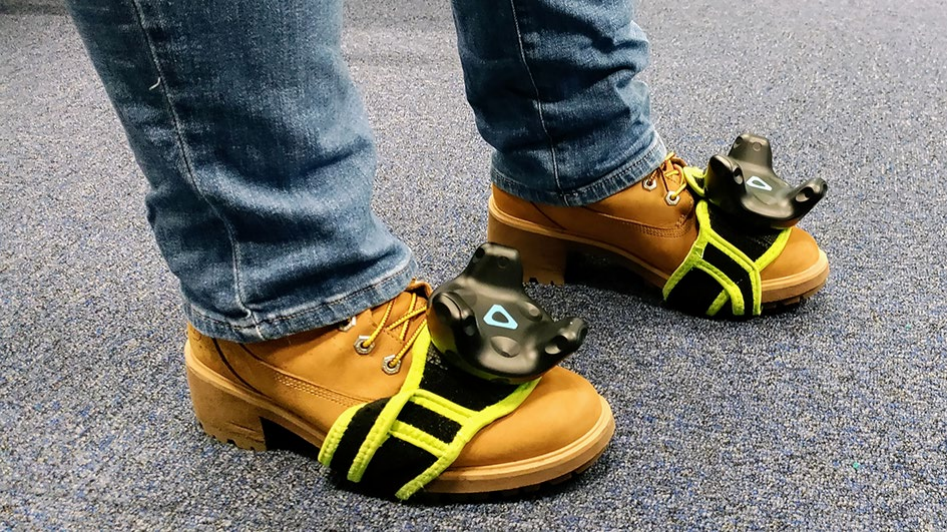
SteamVR trackers applied to feet of participants.

Participants interacted with the blocks via foot movements, which was achieved by attaching sensors to participants’ feet in addition to conventional handheld trackers (Fig. 2). Foot sensors allowed participants to interact with ice blocks in two ways: (1) a one-footed movement to test the block before stepping onto it with both feet, and (2) a two-feet movement in which participants moved fully to the block and stood on it with both feet.

The grid of ice blocks contained three types of blocks. If the block was Solid (green in Fig. 1), it would support the weight of the participant and did not change appearance when activated with either one-foot or two-feet interaction. Crack blocks (blue in Fig. 1) would also support the weight of the participant but any interaction caused a change of colour from translucent to blue accompanied by a cracking sound effect 500 ms after activation. Fall blocks (red in Fig. 1) would not support the weight of participants. A Fall block behaved in exactly the same fashion as Crack blocks during a one-foot interaction, i.e., the block would change colour and make a cracking sound, but any subsequent two-feet activation to the block triggered a shattering sound effect after 500 ms whereupon the block would disintegrate and participants experienced a virtual fall. Therefore, participants learned to use one-footed movements to test each block in order to identify Crack blocks, which could also be the Fall blocks that they should avoid.

Participant psychophysiological measures were recorded simultaneously with a record of their actions within the VE. When the experimenter started the VE, the Unreal Engine application created a text file. As participants interacted with specific virtual objects, such as leaving the Start Study location area or interacting with each block, a time-referenced string value was added to the file. Each interactive ice block was coded with the time the event occurred, interaction type (one-foot or two-footed), block type, and section (see Fig. 1 for section numbers), along with an alphanumeric code (e.g., 12:38:58.089 Intent Solid S1B2). These markers were then used to measure the onset and offset of specific events within the study and to divide psychophysiological measures into discrete events.

The composition of blocks within the VE, specifically the inclusion of Crack and Fall blocks, was configured to manipulate the level of threat experienced by participants (Fig. 1). From section 1 to section 3 (S1:S3), the number of Crack and Fall blocks increase in a linear fashion to acclimatise the participant to an increasing level of threat. The middle sections (S4:S6) is the sole focus of the current study because the threat was maximal, i.e., these three sections contained mostly Crack and Fall blocks, forcing participants to make risky two-footed movements to cracked blocks that could lead to a fall. In the final three sections of the VE (S7:S9), there was a reduction in the number of Crack and Fall threat blocks in a linear fashion.

As participants moved forward through the VE, the previous two rows were removed from behind participants to remove any opportunity for “backtracking”, i.e., participants must keep moving forward (See Fig. 6). In the event of a fall, participants were returned to the block that they occupied before a fall and a gap would appear in the grid to indicate the former position of the Fall block. A video of a participant negotiating the VE is included in the supplementary materials.

### Virtual reality system

**Figure 3:**
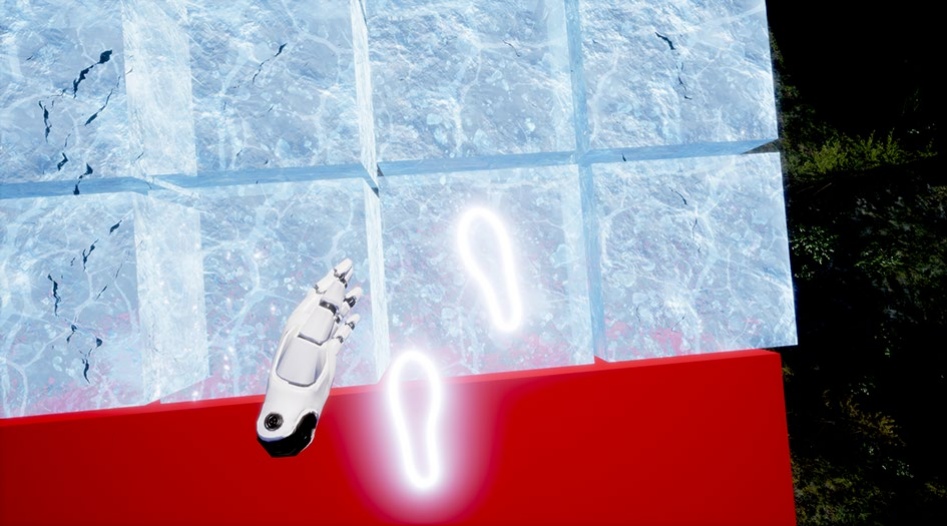
View as solid block is stepped on.

**Figure 4:**
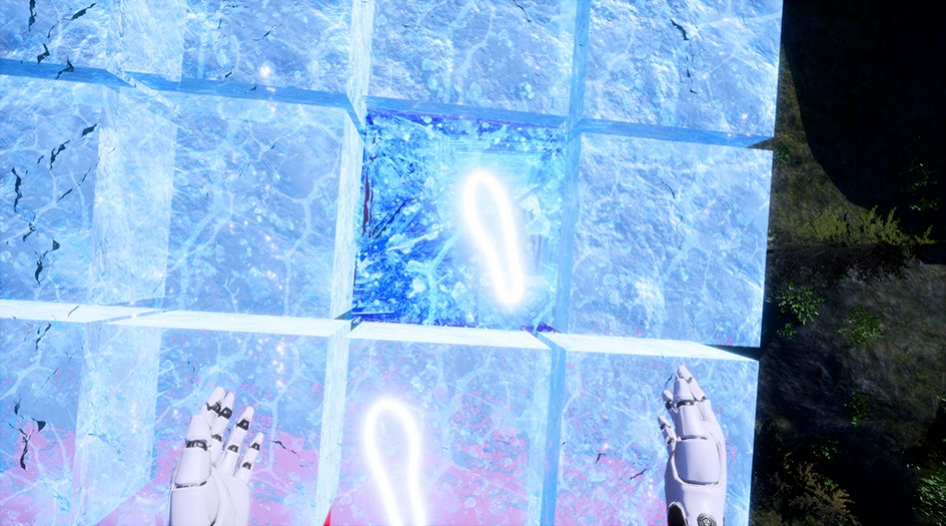
View as crack block is stepped on.

**Figure 5:**
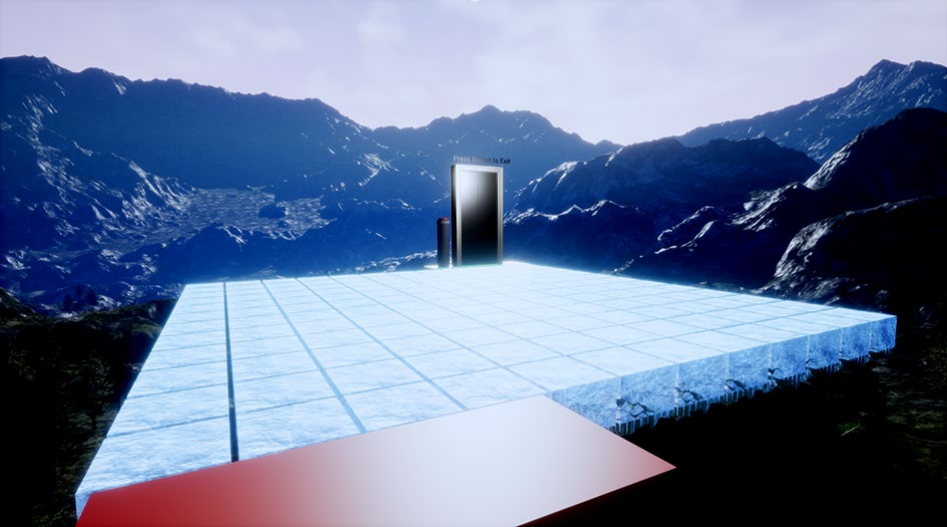
View of environment from starting position (all blocks raised to final height for illustration).

**Figure 6:**
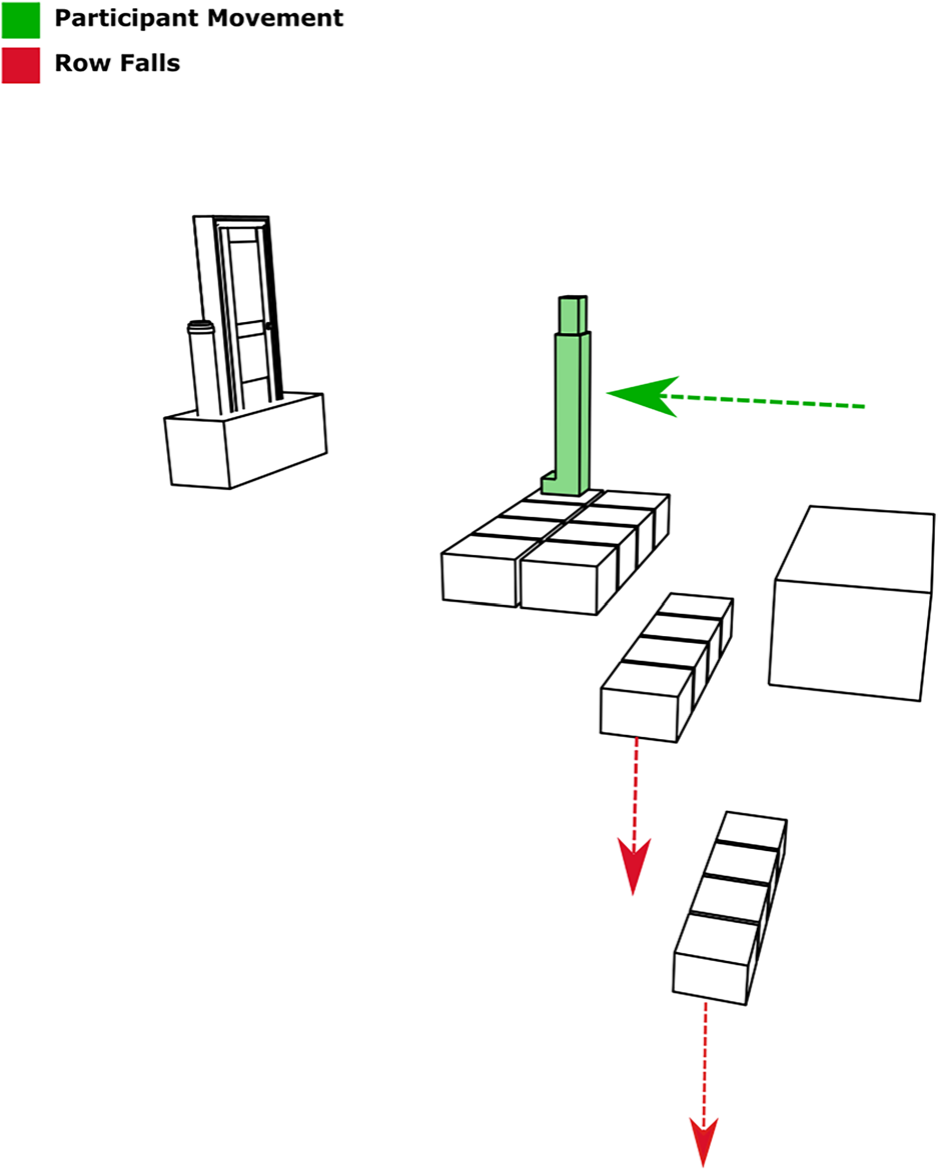
Two rows “falling away” as participant proceeds.

Participants wore a HTC Vive head-mounted display (HMD) with a wireless connection enabled by the HTC Vive Wireless Adapter with four base stations positioned in each corner of the space. Each participant held two hand controllers and two trackers were attached to their feet. Hand and feet positions were represented as robotic hands and white luminous outlines respectively in the VE (Figs. 3 and 4) constructed in Unreal Engine 4.27. All assets were purpose built for the study. The VE was rendered on a desktop PC with custom C++ code integrated directly into the Unreal Engine system to capture interactions with blocks and recorded timings (Figs. 5 and 6).

### Trait impulsivity

Trait Impulsivity was assessed using the eleventh version of the Barratt Impulsiveness Scale (BIS-11)^24^. This questionnaire contains 30 items, which are rated on a 4-point Likert scale that ranges from “Rarely/Never” to “Almost Always/Always.” Responses on the BIS-11 can be used to measure three dimensions of impulsivity, which are: (1) Attentional Impulsivity (i.e., an inability to focus or concentrate), (2) Motor Impulsivity (i.e., a tendency to act before thinking),and (3) Non-planning impulsivity (i.e., a lack of forethought or planning with respect to the long-term consequences of actions).

### Psychophysiology

Skin Conductance Level (SCL) was recorded at 2000 Hz via the Bionomadix ambulatory psychophysiology system (BIOPAC). SCL data were collected from the index finger and second digit of the non-dominant hand and processed in python using the cvxEDA Convex Optimisation to Electrodermal Activity Processing^60^ function to extract the phasic and tonic components of the signal. An electrocardiogram (ECG) was recorded at 2000 Hz via the Bionomadix system with three disposable electrodes placed on the left and right sides of the collarbone and the lower left rib cage. The mean heart rate (HR) was processed in python via the neurokit2 library and the nk.ecg_peaks function. For behavioural data, Time taken in Sections of the VE, one-foot and two-footed movements, markers were generated by the Unreal Engine VE to specify when a participant began and ended the study. These markers were activated either automatically when the study began or via trigger volumes within the VE i.e. touching the virtual button at the end of the task. Markers where also generated via the worn foot trackers during differing interactions i.e. individually for one-footed movements when are participants interacted with one-foot on a virtual block, or in timed beginning and end pairs for two footed decision events i.e. when both feet step on a block and a corresponding end event when both feet stepped off a block.

### Procedure

Participants arrived at the laboratory, they read a Participant Information Sheet and provided informed consent. Participants completed the BIS-11 and received written instructions about the task and the VE. The SCL sensors were taped to the second phalanx of fingers on the non-dominant hand and disposable ECG electrodes attached to both sides of the collarbone and the rib cage. Signal quality of the psychophysiological data were checked. Participants were subsequently fitted with the Vive Tracker sensors on their feet, which were attached to their shoes via velcro-straps. The HMD were placed over the head, adjusted and checked for comfort, and participants received the handheld controllers. The baseline VE was activated and participants were required to stand in a neutral grey toned environment designed to provide no sensory stimulation for a period of 3 min. Participants were instructed to relax during the baseline period. The task VE was activated after this phase and participants stood at virtual height on the starting platform before the first section of 16 ice-blocks (Fig. 1). Participants could progress at their own speed. As they advanced and reached the end row of each the next Section would animate upwards. This design decision prevented rapid advancement and ensured progress into each successive Section preventing advancement to the goal via a direct route, e.g. diagonally across the grid. After advancing through section 9 participants activated the goal doorway and proceeded through it. Participants then had the VR apparatus and psychophysiological sensors removed, were thanked for their time and debriefed.

### Hypotheses and statistical analyses

The VE was divided into three parts (see Fig. 1) to create three levels of threat. In S1:S3, the probability of encountering a crack block rose from 18.75 to 56.25%, with the probability of encountering a fall block rising from zero to 18.75%. This level of threat was sustained in S4:S6. In the final part at S7:S9, the high level of threat from S4:S6 was reduced to 18.75% chance of encountering a crack block and zero probability of a fall block.

We postulated that this behavioural tendency would be maximal during S4:S6 when threat is high and sustained, due to a combination of trait and increased physiological activation. It was also anticipated that any association between subtypes of trait impulsivity and psychophysiology would be most apparent at S4:S6 because physiological activation would be highest when threat is maximal and sustained. Therefore, we only analysed data from S4:S6 in order to focus on the period of peak threat and to minimise the number of statistical tests performed in the analyses.

Five multiple linear regression models were created to explore the association between subtypes of trait impulsivity (independent variables) and dependent variables (time duration, risk ratio, SCL, HR). Each linear regression model was generated using SPSS v.28 (IBM). For all models, normality of residuals was checked and the relationship between normalised residuals and predicted values was visually explored. If any normalised residual or predicted value was greater than 3 or less than − 3, that participant was deemed to be outlier and excluded from the analysis. As six different regression models are reported in the “Results” section, we have used the Bonferroni method to reduce the alpha level from 0.05 to 0.008.

With respect to statistical power, we included five multiple regression models in our analyses, two of which contained 3 predictors, two containing 5 predictors and one containing 4 predictors. Sample sizes for all five models were calculated using G*Power^61^. Because we were using a multiple regression approach where all variables were entered simultaneously, we used the setting for ‘Linear Multiple Regression: Fixed model, R^2^ deviation from zero’ from G*power. The alpha level was set to 0.05 and Power was set to 0.80. With respect to effect size, we anticipated a large effect size (> 0.35) given the strong emotional responses to the VE observed in previous work, we decided to set the effect size to 0.45. We subsequently calculated the minimal sample sizes for the 2-, 4- and 5-predictor models, which were respectively N = 29, N = 32 and N = 35. Therefore, we reached the limit of statistical power for the 5-predictor model using the current sample.

## Results

The analyses of data are reported in two main sections, the first will focus on measures of overt behaviour whereas the second will describe analyses of psychophysiological variables. In both cases, measures of trait impulsivity are utilised as independent variables alongside other covariates to predict dependent variables derived from behaviour and psychophysiology.

Each dependent variable was analysed via a regression model created using data from section 4 to section 6 (S4:S6) when participants experienced a consistently high level of threat.

### Participants descriptive statistics

The study recruited from an opportunity sample and the descriptive statistics for all three dimensions of impulsivity are provided in Table 1 below. In order to illustrate that the average levels of impulsivity for our sample do not deviate significantly from population norms, we have included descriptive statistics in Table 1 from the study conducted by Stanford et al.^62^; their sample of 1577 participants included a large (N = 1178) sample of college students of similar age range to our opportunistic sample.

**Table 1:** Descriptive statistics for impulsivity dimensions of attentional impulsivity, motor impulsivity and non-planning impulsivity for current sample and sample reported by Stanford et al.^62^.

Sample	Statistics	Attentional	Motor	Planning
Current study (N = 34)	Mean	16.1	21.0	24.9
	SD	[3.9]	[4.4]	[4.5]
	Median	15	21	26
	Min	10	11	17
	Max	25	29	34
Stanford et al. (N = 1577)	Mean	16.7	22.0	23.6
	SD	[4.1]	[4.0]	[4.9]

### Behavioural variables

The **number of falls** experienced by participants during the S4:S6 section of the VE was analysed via multiple regression using all three subtypes of trait impulsivity as predictors. One participant was excluded as an outlier. The resulting model did not achieve statistical significance [F(3,29) = 1.29, p = 0.30]. Therefore, none of the three subtypes of trait impulsivity were significantly associated with the recorded number of falls.

The **duration of time** participants spent to complete the S4:S6 section of the VE. The task was self-paced in the sense that participants selected their own speed of movement and the number of 1-footed checks performed prior to 2-footed movements.

The regression model on duration of time for crossing S4:S6 was significant [F(3,27) = 3.33, *p* = 0.03] with a R^2^ of 0.27 (Adj-R^2^ = 0.21). The coefficients are presented in Table 2, which show that higher levels of non-planning impulsivity were significantly associated with shorter time duration to complete S4:S6.

**Table 2:** Coefficients in the regression model for duration of time to complete S4:S6.

Coefficient	Standard beta	t	Sig	Tolerance	VIF
Attentional	0.27	1.43	.163	0.76	1.31
Motor	− 0.35	1.75	.090	0.70	1.44
Non-planning	− 0.55	− 2.84	.008	0.71	1.41

This model revealed that non-planning impulsivity had a significant negative relationship with time to complete the section, i.e., individuals with higher non-planning impulsivity moved faster across S4 to S6.

The level of **risky behaviour** exhibited by participants as they negotiated the VE was calculated by creating a ratio measure based on the proportion of 2-footed movements to one-footed checks. When confronted with an ice block, participants had the option of checking each block with one foot (to check if it was a cracked block) before choosing to step onto the block with both feet. Therefore, our risk ratio represents the number of two-footed movements divided by the number of one-footed checks, i.e., an increase of the risk ratio = smaller number of one-footed checks: 2-footed movements. We reasoned that the frequency of falls experienced by participants would also influence their level of behavioural risk and included falls in the three regression models alongside the impulsivity subtypes. Four participants were removed from this model as outliers. The R^2^ for this model was 0.35 (Adj-R^2^ = 0.27) and it was statistically significant [F(4,25) = 3.39, p = 0.02], see Table 3 for listing of coefficients.

**Table 3:** Coefficients in the regression model for the risk ratio measure.

Coefficient	Standard beta	t	Sig	Tolerance	VIF
Attentional	− 0.04	− 0.23	.819	0.73	1.38
Motor	− 0.18	− 0.89	.382	0.67	1.52
Non-planning	0.55	2.97	.006	0.75	1.33
Frequency of falls	0.39	2.31	.029	0.91	1.01

The analysis of risk ratio indicated that participants with higher levels of non-planning impulsivity had a higher score on the risk ratio, i.e., they made a smaller number of one-footed checks compared to two-feet movements.

### Psychophysiology

**Skin Conductance Level (SCL)** was captured for each third of the VE and was baselined for each participant. The mean baseline-adjusted SCL (henceforth called mean SCL) was entered into a regression model as a dependent variable with trait impulsivity as independent variables. Because speed of movement and frequency of falls could also influence mean SCL, both those independent variables were added to the regression model. Four participants were excluded as outliers from this model.

The regression for mean SCL during S4:S6 was found to be significant [F(5,24) = 5.41, p < 0.01] with a R^2^ of 0.53 (Adj-R^2^ = 0.47). The coefficients for this model are presented in Table 4.

**Table 4:** Coefficients in the regression model for SCL.

Coefficient	Standard beta	t	Sig	Tolerance	VIF
Attentional	− 0.09	− 0.54	.594	0.79	1.27
Motor	− 0.42	− 2.23	.035	0.55	1.83
Non-planning	0.60	3.47	.002	0.65	1.54
Frequency of falls	0.49	3.28	.003	0.87	1.14
Movement time	0.10	0.63	.535	0.78	1.28

The coefficients in the regression model revealed a significant positive relationship between mean SCL and both frequency of falls and non-planning impulsivity, i.e., higher mean SCL for participants with a higher number of falls and higher scores on non-planning impulsivity.

**Heart rate** data (beats-per-minute) was collected from participants during a one-minute long standing baseline and during the VE. A baselined mean Heart Rate (HR), henceforth called mean HR, was calculated for S4:S6 and used as a dependent variable in combination with the same independent variables that were entered during the analyses of mean SCL. However, the resulting model was not statistically significant [F(5,28) = 0.34, *p* = 0.88].

## Discussion

One purpose of the study was to explore the relationship between impulsivity subtypes and behaviour during changing levels of threat in VR. Our analyses revealed that participants’ behavioural and psychophysiological responses to high levels of threat in the VE were significantly influenced by their scores on the non-planning impulsivity subscale of the BIS-11. Our analyses revealed a positive association between the mean level of skin conductance level, which is a marker of sympathetic activation, and non-planning impulsivity (Table 4). From a behavioural perspective, those with high scores on non-planning impulsivity also travelled from S4 to S6 in less time (Table 2) and with a reduced frequency of one-footed checks before the decision to move to a new block (Table 3).

The association between non-planning impulsivity and elevated SCL revealed by our analyses is supported by earlier work^44^ where impulsivity was linked to greater autonomic reactivity under conditions of challenge for this subtype of impulsivity. Based on our analyses of SCL and heart rate, we found no evidence to support the underload hypothesis^38^ i.e., reduced autonomic reactivity to threat for participants who score high on trait impulsivity. This is notable because the level of threat and likelihood of a fall was both high and sustained through S4 to S6 (Fig. 1).

Non-planning impulsivity was associated with lower completion time and risky behaviour, e.g., higher ratio of two-footed to one-footed interactions, which was indicative of fewer one-footed checks before stepping on blocks with both feet (Tables 2 and 3). Non-planning impulsivity was also positively associated with SCL (Table 4). These findings beg a number of questions with respect to the direction of causality. Did a strategy of fast movement/little forethought/reduced checking increase physiological activation because non-planning participants negotiated the VE in a way that was fast, ill-considered and inherently risky? Or were the higher levels of SCL observed for those participants reflective of a more intense emotional response to threat that created the impetus for them to behave in a risky way? We also observed a positive association between the frequency of falls and elevated SCL (Table 4), which could also be contribute to the effect observed for individuals with higher scores on non-planning impulsivity; however, we found no evidence of a significant association between falls and scores on any subscales of impulsivity, i.e., scoring higher on non-planning impulsivity, despite a style of moving across S4:S6 that was inherently risky, did not result in a greater number of falls. It could also be argued that greater physiological activation resulted from higher metabolic demands of faster movement speed, however both speed of movement and trait non-planning impulsivity were entered in the same regression model, and their respective effects on physiological activation appeared to be independent of one another.

With respect to psychological explanations for our primary finding, it could be argued that non-planning impulsivity is associated with greater physiological activation as a consequence of a tendency to seek risk^24,25^. Alternatively, we can adopt a perspective from the somatic marker hypothesis^40^ wherein participants with high non-planning impulsivity exhibited greater physiological activation but perhaps lack sufficient interoceptive sensitivity^45^ to integrate these ‘danger signs’ into their decision-making and subsequent behaviour. We could also reverse our direction of inference regarding the relationship between non-planning impulsivity and behaviour/physiology. If individuals with high scores on non-planning impulsivity represent a tendency towards a lack of forethought with respect to long-term consequences of current actions, then those individual with low scores on this trait are predisposed to behave in the opposite direction, i.e., to give significant thought to the consequences of their actions. Therefore, individuals with low scores on non-planning impulsivity perform a greater number of checks in advance of action, as a consequence they tend to move more slowly. Because these individuals move slowly in a methodical and considered way, they are less sympathetically activated than their peers with a greater tendency towards non-planning impulsivity. The combination of linear modelling with bipolar traits as dependent variables makes it difficult to draw strong conclusions about whether low or high scores on the trait are driving the observed experimental effect. One solution to this ambiguity is to opt for a stratified sampling strategy wherein participants are specifically recruited into three groups: high trait scores, low trait scores, and trait scores that align with the population average. This recruitment can be combined with inter-groups comparisons via ANOVA or MANOVA, which differentiate where low and high scores on trait impulsivity significantly deviate from the population norm.

The primary weakness of the current work was the limited sample size, which was less than ideal for studying individual differences with a convenience sample and an acknowledged weakness with respect to statistical power for the models of psychophysiology. In defence of the current study, a previous study using the same VE found that a similar N was sufficient to uncover statistically significant differences in behaviour between high and lower scorers on trait neuroticism using a median split approach^59^. While the sampling approach for the study was not structured, there is some evidence that our participants’ scores on the trait impulsivity subscales did not deviate substantially from a larger population (Table 1). However, it is acknowledged that our results must be regarded as tentative and based on a limited sample, and in need for replication with a larger sample and across different experimental tasks. In addition, the current study relied heavily on self-reported levels of trait impulsivity and the BIS-11 in particular. Trait impulsivity can be measured using other self-report tools^24^ and previous research^63,64^ does not always support the three-factor analysis of subtypes used in the current study. It is also possible to measure trait impulsivity using standardised behavioural tests^65^ either to compliment self-report scales or as an alternative to their use. While the current study revealed differences between impulsivity subtypes and behaviour/physiology, it would be ideal to replicate our findings using a multimodal range of measures to capture subtypes of trait impulsivity.

The purpose of the VE was to induce threat in a way that fully engaged the participants, and most importantly, provided them with enough freedom to respond to threat in different ways. However, the.

VE could be improved to promote autonomy and strategic thinking by perhaps reducing the number of crack blocks and designing a layout with a more finely-tuned manipulation of threat. Alternatively, our approach may be improved by adopting an experimental design wherein participants negotiate different layouts of the VE where the level of threat is stable throughout; exposure to different layouts of the VE could be accommodated into either a within or between-participants design. Additionally, the VE design could be adapted to increase its leverage over programmatic mechanisms made available by Unreal Engine. The ‘S’ shaped course of the study (See Fig. 1) led to transition points between Sections 3 and 4 and Sections 6 and 7 which reduced immersion and interactions with blocks which were not consistent with the rest of the task. The task could be redesigned to follow a circular pattern that could be exponentially ‘longer’. This would increase the number of block interactions and allow the task to more gradually modulate the level of threat experienced by the participants.

Our methodology demonstrated the potential of using a room-scale VE to elicit realistic behaviour, induce strong emotional responses to threat and permit our participants a sufficient level of agency so we could observe individual differences. The design of the VE, in this case the layout of the blocks shown in Fig. 1, also permitted a high level of control over our key independent variable (threat). By combining measures from psychophysiology with the tracking of movement, we were able to explore both physiology and behaviour within the same statistical models. This approach could be developed by expanding the range of sensors incorporated into the study and increasing the fidelity of behavioural monitoring. For example, individual decisions about which block to step onto with two feet could be analysed on an event-related basis with respect to behaviour, psychophysiology and neurophysiology.

To summarise, the current study provides some evidence that subtypes of trait impulsivity can be identified with respect to changes in physiological activation and behavioural choices in response to threat within a virtual environment.

### Supplementary Information

Supplementary Information 1.

Supplementary Information 2.

Supplementary Information 3.

## Data Availability

The datasets used and/or analysed during the current study available from the corresponding author on reasonable request. All data generated or analysed during this study are included in this published article and its supplementary information files.
