# Supplementary material for: Trait impulsivity influences behavioural and physiological responses to threat in a virtual environment
Source: Sci Rep. 2024 Apr 25;14:9484. doi: 10.1038/s41598-024-60300-6 (PMC11045749; doi:10.1038/s41598-024-60300-6)
Supplement: Supplementary file 2 — Supplementary Information 2. [file 41598_2024_60300_MOESM2_ESM.docx]

Supplementary Video 1: Trait Impulsivity Threat VR Environment
